# Ubiquitin-Dependent and Independent Proteasomal Degradation in Host-Pathogen Interactions

**DOI:** 10.3390/molecules28186740

**Published:** 2023-09-21

**Authors:** Wojciech Bialek, James F. Collawn, Rafal Bartoszewski

**Affiliations:** 1 Department of Biophysics, Faculty of Biotechnology, University of Wrocław, 50-383 Wrocław, Poland; 2 Department of Cell, Developmental, and Integrative Biology, University of Alabama at Birmingham, Birmingham, AL 35233, USA; jcollawn@uab.edu

**Keywords:** ubiquitin, host–pathogen interactions, 26S proteasome, 20S proteasome, protein degradation, E3 ligases, deubiquitinases, UPS

## Abstract

Ubiquitin, a small protein, is well known for tagging target proteins through a cascade of enzymatic reactions that lead to protein degradation. The ubiquitin tag, apart from its signaling role, is paramount in destabilizing the modified protein. Here, we explore the complex role of ubiquitin-mediated protein destabilization in the intricate proteolysis process by the 26S proteasome. In addition, the significance of the so-called ubiquitin-independent pathway and the role of the 20S proteasome are considered. Next, we discuss the ubiquitin–proteasome system’s interplay with pathogenic microorganisms and how the microorganisms manipulate this system to establish infection by a range of elaborate pathways to evade or counteract host responses. Finally, we focus on the mechanisms that rely either on (i) hijacking the host and on delivering pathogenic E3 ligases and deubiquitinases that promote the degradation of host proteins, or (ii) counteracting host responses through the stabilization of pathogenic effector proteins.

## 1. Introduction

The proteasome, a key regulator of protein homeostasis, accounts for as much as 1–2% of the proteome in healthy cells [1]. The proteasome not only degrades misfolded or otherwise damaged proteins [2], but also works together with the autophagy machinery to dispose of larger protein aggregates, intracellular bacteria, or even organelles [3]. By maintaining intracellular protein quality control, it regulates many aspects of cellular existence, including the cell cycle, apoptosis [4], and antigen processing [5]. While the proteasome’s age-related reduction in activity may lead to neurodegeneration [6], its role in inflammation-related diseases and cancer [7] is well-characterized, and has prompted the development of proteasome-specific drugs [8].

Initially, proteasomal degradation was intimately linked to the post-translational modification of ubiquitination. This specific modification relies on the attachment of one ubiquitin (Ub) or several ubiquitin residues to the designated substrate. The reaction is orchestrated by a set of three enzymes: E1 responsible for ATP-dependent Ub activation, followed by the transfer of a Ub thioester to a Ub-conjugating enzyme, E2, and finally, E3, catalyzing an isopeptide bond formation. The Ub chain can be elongated through the successive attachment of Ub moieties to the N-terminal methionine residue or, more frequently, to a lysine side-chain present in Ub (K6, K11, K27, K29, K33, K48, K63), resulting in different forms of linear or branched polyUb chains [9]. In fact, the type of assembled polyUb chain determines the final fate of the substrate. This varies from the modification of its activities to the modulation of protein localization or interactions [10], or the regulation of its half-life (which ranges from seconds to hours). Consequently, the Ub system is very diverse.

For years, the precise role of the Ub tag remained elusive. It was initially regarded as a recognition tag, but later, with the advent of X-ray and Cryo-EM [11,12] high-resolution structural studies that were supplemented with single-molecule FRET analysis [13], more light was shed on this topic. It was revealed that specific interactions between Ub substrates and the proteasome result in conformational changes regulating the target’s fate. Here, we provide a concise summary of the latest developments in the ultimate function of the Ub tag in the process of protein degradation. Furthermore, we discuss how animal and plant pathogens manipulate eukaryotic proteasomal degradation, to their benefit, in a varied and sophisticated manner.

## 2. Ub-Mediated Proteasomal Degradation

Eventually, every single protein, depending on its lifetime, is destined for degradation. Misfolded or damaged proteins must be swiftly recognized and destined for recycling in order to prevent the toxic buildup of aberrant proteins. Their primary means of disposal is through the concerted action of E1, E2, and E3 enzymes, ubiquitination, and proteasomal degradation. This protein quality-control mechanism is termed the ubiquitin–proteasome system (UPS) [3].

In eukaryotic cells, the proteasome functions in both the nucleus and cytoplasm [14]. It exists as the 26S holoenzyme, composed of the 19S regulatory particle, which is responsible for recognizing the Ub degradation tag and unfolding the target polypeptide–substrate, and the 20S catalytic core that proteolytically cleaves the unfolded protein [15]. Since the detailed three-dimensional structure of the 26S has been reviewed recently [16], here we only highlight the features that are essential for the scope of this review. 

To understand how pathogens escape proteasomal degradation or induce degradation of the host proteins, we first need to delve into the molecular mechanisms of the proteasomal machinery. Regarding the degradative functions of the 26S proteasome, its Ub-binding domains bind various unrelated proteins whose only common denominator is the specific conjugated Ub chain. This megacomplex can recognize delivered proteins through its Ub receptors, Rpn1, Rpn10, and Rpn13 [17,18,19]. The Ub moieties are disassembled from the substrate at a later stage by proteasome-associated deubiquitinase (DUB), Rpn11 [20]. However, other DUBs, namely USP14 and UCHL5, bind transiently to the 19S to facilitate the degradation process. Notably, instead of being a non-selective molecular machine, the proteasome itself can decide whether to degrade a substrate or prolong its lifetime by deubiquitinating it [21]. Interestingly, K48-linked tetraUb is remarkably resistant to deubiquitination, highlighting their role as an efficient degradation trigger [22]. Disassembled Ub molecules can eventually be recycled and attached to another target molecule.

Importantly, in addition to simply targeting proteins for the proteasome, Ub chains can also activate peptidase [23,24], ATPase [25], and unfolding [26] activities. The proteasomal Ub receptors were proposed as a trigger of these activities, and it was shown that polyubiquitinated substrates interact with Ub receptors on the 19S regulatory particle of the proteasome. Even though Ub chains are disassembled before unfolding and degradation can occur, they directly govern the unfolding of a target protein. The three-dimensional structure of the Ub–substrate conjugate determines which receptors are engaged for efficient unfolding. In some cases, the simultaneous involvement of multiple Ub receptors may be required. Although the three intrinsic proteasomal Ub receptors, Rpn1, Rpn10, and Rpn13, are involved, and Rpn13 plays the most prominent role, there is also some redundancy between these receptors [27]. 

The 26S proteasome recognizes thousands of protein substrates through the attached long Ub chains and uses its ATP motor for mechanical unfolding and translocation into a proteolytic chamber. Despite the application of state-of-the-art methodologies such as Cryo-EM, only the proximal Ub moieties could be initially identified. It seemed likely, however, that Ub within the chain dynamically interacts with multiple receptors in the regulatory particle. In fact, Ub chains allosterically regulate degradation initiation by affecting the rates of switching between functionally distinct 26S proteasome conformations [28].

Before being degraded by the proteasome, many ubiquitinated proteins must first be extracted from macromolecular complexes and membranes using a specific unfoldase, (Cdc48 in yeast and p97/VCP in mammals). Cdc48 is a homohexameric AAA ATPase that mediates the unfolding of Ufd1/Npl4-recruited substrates by pulling on the proximal Ub and moving all Ub molecules linked to its C-terminus through the central pore of the hexameric double ring [29]. An unexpected observation was that this leads to the transient unfolding of Ub, which is otherwise known for its unparalleled stability [30]. Nevertheless, this study showed, for the first time, that a groove in Npl4 is poised to bind unfolded Ub, and that the unfolded Ub is actually threaded into the central pore of the Cdc48 core.

Multiple Ub chains on a single target protein can enhance binding affinity to proteasomes even though a single K48 tetraUb chain sufficiently triggers substrate degradation [31]. However, by simultaneously activating numerous Ub receptors or DUBs, the extra Ub chains strengthen the commitment to substrate degradation [32]. Interestingly, there is a fundamental difference between yeast and mammalian 26S proteasomes. While only about five Ub moieties are enough to unfold ubiquitinated proteins by yeast Cdc48-Ufd1-Npl4, the p97 form requires either much longer chains or accessory proteins, namely UBXN7, FAF1, or FAF2, which reduce the Ub threshold requirement of the p97-UFD1-NPL4 accessory proteins [33]. This also allows for the fine-tuned regulation of the degradation process, which can also be achieved by another means, such as through the utilization of K11/K48-branched Ub chains that are particularly efficient at promoting degradation [34]. However, in some cases, heterotypic Ub chains may increase the stability of the modified proteins [35]. 

Other intriguing features of the Npl4–Ufd1 cofactor complex were revealed when it was shown that Npl4–Ufd1 recognizes substrates decorated with K48-linked polyUb chains [36,37]. This specificity between Npl4–Ufd1 and three Ub moieties is achieved by recognizing unique conformation characteristics for K48 linkages. It was also suggested that an additional Ub-binding domain could bind another distal Ub moiety, and this fourth Ub-binding site might capture various Ub moieties at the distal end of a chain [38]. 

The latest discoveries shed even more light on the mechanisms and kinetics of the whole process of Ub unfolding, its insertion into the ATPase pore, and the unfolding of the ubiquitinated substrate. The Ufd1 protein has a specific domain, called UT3, that acts as a sensor for the type of linkage between the Ub molecules in the chain. The linkage specificity of the UT3 domain allows for the Ufd1/Npl4 complex to selectively engage polyUb chains. The processing of the polyUb chain, specifically the removal of Ub molecules that are distal to the substrate, is rate-limiting for the release of the substrate [39]. Ub engagement and translocation, as well as the unfolding of the attached substrate protein, occurs in 2–5 s, whereas the release of the Ub substrate takes about 2 min [39]. Since the Ufd1’s UT3 domain is a proximal Ub sensor that determines the linkage specificity of Cdc48-UN, this information can be used to help design drugs that target specific Ub linkages.

## 3. Specific Requirements for Efficient Proteasomal Degradation

Virtually all post-translational modifications, such as phosphorylation or acetylation, change the biophysical properties of the modified protein. Although Ub was initially regarded as a recognition tag, it was speculated that the attachment of such a large molecule to the target protein might significantly alter the properties of the conjugate. Indeed, earlier studies have shown that ubiquitination affects the solubility and refolding [40], stability [41], and conformational dynamics [42] of the modified proteins. In fact, in the case of multidomain proteins, which resemble ubiquitinated proteins to some extent, it is known that individual domains interact with each other and affect the folding of the whole molecule [43]. Thus, questions arose about the significance of these Ub-induced alterations in the cellular context. 

Given that the proteasome must be able to process numerous structurally different substrates efficiently, including well-folded, partially folded, and disordered proteins, it has been suggested that ubiquitination itself may enhance the substrate’s unfolding capacity to assist in the degradation process. Indeed, the attachment of Ub may directly affect the substrate’s biophysical features in addition to its signaling role. An in-depth analysis has shown that ubiquitination strongly affects thermodynamic stability, with the outcome depending on the type of Ub moiety and the position of the ubiquitination site on the substrate. In the case of Ubc7, ubiquitination at residues that are targeted in vivo for the attachment of K48 chains results in thermal destabilization and a local unwinding near the modification site [44], ultimately leading to proteasomal degradation. 

In other cases, the K48 ubiquitination of alternative sites, i.e., those not targeted by the machinery, resulted in various outcomes. These range from strong stabilization to no effect or varied degrees of destabilization. The authors suggested that the site where ubiquitination occurs in vivo may be evolutionarily selected to ensure an efficient unfolding process. Moreover, the other types of ubiquitination, such as monoUb and K63-Ub, did not lead to such a strong destabilization, as evidenced for K48-chains. The fact that a K48-polyUb affects the substrate in a specific manner indicated that it may have evolved for this specific function. In addition, the authors emphasized a synergetic effect of ubiquitination and phosphorylation on the degradation process via enhanced thermal destabilization [44]. Of note, this phosphorylation–ubiquitination cross-talk has also been highlighted in many studies on the ubiquitination of oncoproteins [45]. 

Ubiquitination sites involved in degradation can be found in both structured and disordered regions [46]. However, ubiquitination at a non-destabilizing site prevents efficient proteasomal degradation. Apart from the attached Ub, a substrate destined for the proteasome must contain an unfolded or partially unfolded disordered region [47]. Only the presence of both a Ub-attached moiety to a substrate and the unstructured region allows for rapid degradation rates by the 26S proteasome. In contrast, the rapid proteolysis of a non-ubiquitinated form of the same protein appears to be a signature of the 20S proteasome [48]. This two-factor safety measure controls the proteasome activity and keeps it in check [49]. 

While the typical signal for degradation is the attachment of a polyUb chain, one can assume that the probability of the ubiquitination of all surface-exposed lysines is the same. Furthermore, it is tempting to assume that E3 ligases alone determine the specific lysine residue that is to be modified [50]. That being said, several modes of target recognition have been proposed that require the involvement of other binding partners, post-translational modifications, and specific E2 enzymes (reviewed in [51]). Indeed, numerous E3s are highly specific towards substrates and sites of ubiquitination, whereas other E3s are highly promiscuous [52]. 

Some E3s, especially those classified as Cullin–RING ligases (CRL), can target virtually all lysine residues present in a so-called ubiquitination zone in a rather nonspecific manner [53,54]. One of the most studied examples is APC/C. Detailed studies actually show some specificity towards lysines that flank serine residues on disordered degrons [55]. These degrons are short N- and C-terminal degradation motifs that determine the half-life of a protein (comprehensively reviewed in [56]). Furthermore, ubiquitination induces the substrate’s unfolding in a site-specific manner and allows for the substrate to access high-energy states only when modified at certain sites [57]. Subsequently, it was also shown by the same group that the proteasome selectively recognizes and degrades substrates ubiquitinated at these destabilizing sites [58]. Altogether, these landmark studies reveal a new layer of regulation for proteasomal degradation.

Recent studies indicate that the role of attached Ub extends far beyond mere proteasomal recruitment. Another landmark report provided evidence that ubiquitination destabilizes the folding of two proteins, FKBP12 and FABP4, and that elongation of the conjugated Ub chains further enhances this destabilization effect. In this study, NMR relaxation analysis provided evidence of the impact of ubiquitination on protein backbone dynamics and intrinsic protein motion. The larger values and deviations of J(0) observed for ubiquitylated proteins suggested that fluctuations in the protein backbone contribute to the observed structural changes [28]. The degree of destabilization is more severe when ubiquitination occurs in a β-sheet compared to when it occurs in a loop region or in an α-helix. Furthermore, ubiquitination-related structural fluctuations are not exclusive to the ubiquitination site but are distributed rather globally, even in the case of a multi-domain protein such as calmodulin [28]. In summary, ubiquitination-induced fold destabilization correlates with the ubiquitination site location in the substrate protein and the related secondary structure elements that determine the degree of destabilization.

## 4. Ub-Dependent versus Ub-Independent Protein Degradation

Overall, the presence of intrinsically disordered regions explains the susceptibility of some proteins, especially intrinsically disordered proteins (IDPs), to proteasomal degradation. These proteins differ in many ways from folded proteins. For instance, while the attachment of Ub reduces the thermal stability of folded proteins, thermotolerant IDPs are readily processed by 20S proteasome [59]. In addition, it is clear that Ub is not absolutely necessary for substrate degradation in some cases. The degradation of the intrinsically disordered protein tubulin-associated unit (Tau) by the 20S proteasome occurs in an Ub-independent manner. The cleavage of Tau by the 20S proteasome is most efficient within the aggregation-prone repeat region and generates both longer fragments and short ones, which are aggregation-deficient peptides [60]. 

A proteomic approach led to the identification of ~500 IDPs substrates of the 20S proteasome, which is now known to degrade IDPs via an Ub-independent, disorder-driven mechanism. These 20S proteasome substrates were highly disordered and enriched for RNA binding proteins, particularly those involved in splicing, mRNA processing, and translation [61]. Consequently, the authors concluded that the so-called 20S-IDPome is significantly more disordered than the human IDPome. In particular, this specific subset includes low-complexity proteins with prion-like domains, proteins involved in miRNA biogenesis, and those implicated in ALS disease [61].

Notably, further studies identified proteins that can be degraded by both Ub-dependent and Ub-independent pathways [62,63]. In this case, depending on whether or not the substrate is ubiquitinated, the proteasome can switch between ATP-dependent robust unfolding and weak ATP-independent degradation. Furthermore, proteins that contain N-terminally disordered regions, such as the human cyclin B1, may be a substrate for both 20S and 26S proteasomes. However, 20S complexes are more efficient than 26S in degrading such a native disordered protein [64] due to the presence of one of the 20S proteasome subunits, PSMA3, which preferentially interacts and traps IDPs [65]. Indeed, unmodified cyclin B1, i.e., a Ub-free and intrinsically disordered protein, is readily degraded by the 20S proteasome [48]. 

Conversely, a chemically synthesized panel of well-defined homogenous substrates showed, as expected, that the longer the K48 chains that attached to cyclin B1, the faster the protein is degraded by purified 26S proteasomes in vitro. By binding to the Ub receptors, the Ub moieties facilitated the degradation of a tagged substrate by the 26S proteasome, which was not the case for the 20S proteasome that lacks Ub receptors. Unlike 26S, which recycles Ub moieties, 20S surprisingly proteolyzes both the Ub attached to the substrate and the unstructured conjugate. In addition, the authors of this seminal work show that, under hypoxia, the 20S proteasome degrades damaged proteins and improves cell viability [48]. It was concluded that the 20S proteasome exhibits a signature behavior distinct from that of the 26S proteasome and that this ability could be beneficial during stress.

Interestingly, the degrons mediating Ub-independent proteasomal degradation in cells are transferrable, and this was demonstrated by fusing the degron to an otherwise stable bacterial protein, BirA [66]. In fact, Ub-independent degrons are found in several proteins, including mammalian thymidylate synthase [67], yeast Rpn4 [68] and ornithine decarboxylase [69]. This tag provided a mechanism for proteasomal targeting without the need for ubiquitination. In these cases, this degradation is less robust overall, with complete degradation only occurring with loosely folded substrates [70]. This indicated that the proteasome can only capture these substrates if they are transiently unfolded. 

The latest advances in the field have shown that Ub-independent proteasomal degradation is not limited to a set of unusual proteins, as previously believed. In contrast, together with the classical Ub-dependent pathways, Ub-independent mechanisms also execute the protein-quality functions of proteins controlling cell proliferation and survival such as REC8 and CDCA4 [66], and likely many other proteins that have yet to be identified.

The specific Ub-chain attached to the substrate impacts the proteasome’s capacity to unfold. The substrates that were ubiquitinated by the Keap1/Cul3/Rbx1 E3 ligase complex, which produced mixed-linkage chains containing both K48- and K63-linkages, had a higher capacity for unfolding than those that were ubiquitinated by Rsp5, which produced exclusively K63-linked chains. Higher unfolding capacities were achieved by either ubiquitination method compared to substrates that were delivered to the proteasome via Ub-independent degrons [26].

In general, unfolding requires ATP, but ATP, as an essential component for the proper functioning of the 26S proteasome, has other distinct functions, such as maintaining stability and promoting Ub processing. Theoretically, the presence of ATP may not be necessary for the degradation of certain proteins that are not ubiquitinated and do not require unfolding. In fact, physiological concentrations of NADH can replace ATP [71], and the NADH-stabilized 26S proteasome is efficient in degrading IDP substrates that do not require ATP-dependent unfolding, such as p27, Tau, and c-Fos [72]. This would apply to proteins containing IDRs or a bona fide IDP, and this constitutes about one-third of the eukaryotic proteome. Many of them were shown to be degraded in an ATP-independent manner by the 20S proteasome [73].

A subset of proteins that are inherently unstable and degraded “by default” by the 20S proteasomes exists in cells. These substrate proteins, however, can be protected by interactions with each other or with other proteins, or even upon their assembly into large functional protein complexes. For instance, the tumor-suppressor proteins p53 and Rb, which are targeted by different E3 ligases for Ub-dependent 26S proteasomal degradation, can be redirected toward Ub-independent 20S proteasomal degradation [74]. In fact, both p53 and p73, and likely other proteins, can also avoid 20S degradation upon binding to NQO1 in an NADH-dependent manner [75]. Similarly, PGC-1α, another IDP and a key metabolic regulator, can be protected from the 20s proteasome by NQO1, which could be designated as a “professional stabilizer” [76].

## 5. Human Pathogens Hijacking the UPS

Given the sheer number of functions that UPS plays in cells, it comes as no surprise that its dysregulation presents a danger to cells. Whereas both the non-proteolytic ubiquitination in signaling and in human disease [77] and proteolytic ubiquitination in tumorigenesis and cell cycle control [45] are most often reviewed, we report here that host–pathogen interactions that occur through the UPS have been gaining attention in recent years. Remarkably, it is widely accepted that pathogenic bacteria and viruses lack their own Ub system, but instead can designate proteins with a prokaryotic ubiquitin-like protein (Pup) for proteasomal degradation, at least in some cases [78]. Nevertheless, they have mastered the ability to hijack host ubiquitination machinery for their own benefit, including disabling key targets, evading the cell defense system, and promoting their replication and pathogenicity [79]. Although bacteria have evolved several different sophisticated methods to promote their proliferation through the host Ub system, including modifying the dynamics of the actin cytoskeleton [80] or impairing JNK’s activation [81], here we focus on the strategies related directly to protein stability (summarized in Table 1). 

All cellular compartments are vulnerable to pathogen infection, starting from the very first step of entering the cell. Some viruses rely upon ubiquitination, which promotes general internalization and sorting to the late endosomes and lysosomes [82]. The influenza A virus (IAV) utilizes a combination of viral and cellular mechanisms to coordinate the transport of its proteins and gene segments in and, when fully assembled, out of the cell. During uptake, the viral ribonucleoprotein complex is released from the endosome by taking advantage of a host E3 ligase, Itch. Itch ubiquitinates the viral protein M1, triggering viral egress into the cytosol and eventually transporting it to the nucleus [83], where it replicates.

There has been much research surrounding the concept of pathogens compromising the host immune response. This problem has always attracted considerable attention and the discovery of pathogens’ ability to hijack the UPS has shed even more light on this topic. As the list of host proteins targeted for proteasomal degradation is still growing, here we illustrate the ways in which the pathogens inhibit antigen presentation. 

Antigen presentation is crucial for triggering T cell immune responses. Two classes of major histocompatibility complexes (MHC), MHC-I and MHC-II, are involved in this process, and both of them are attractive targets for pathogens. Upon ubiquitination, MHC-II is internalized and directed towards endolysosomal degradation while resulting peptides are presented at the cells surface as antigens. The MHC-II -related antigen presentation is downregulated by the Salmonella effector SteD [84]. This depletes mature MHC-II molecules from the surface of infected antigen-presenting cells through their E3 ligase activity, which results in MHC class II ubiquitination and degradation [84]. To enhance its effect, SteD is also ubiquitinated, leading to even more reduced levels of surface MHC-II [84].

Some amino acid sequences serve as stop signals for proteasomal degradation. The Gly-Ala repeat, found in the Epstein–Barr virus protein EBNA-1, prevents EBNA-1 proteasomal degradation [85]. This is required for the generation of EBNA-1 peptides that MHC-I can use to bind, present, and activate cytotoxic T-cells. Introducing the Gly-Ala sequence into EBNA-4 chimeras [86], as well as Gly-Ala p53 functional chimeras, allows these chimeras to efficiently escape proteasomal degradation by inhibiting their unfolding [87], despite the fact that they are ubiquitinated [88]. Nevertheless, the presence of these repeats alone cannot fully explain EBNA-1’s long stability in cells [89]. The authors also noted that, when the Gly-Ala repeat is placed in close vicinity to other unfolded regions, the 26S proteasomal degradation of the protein was inhibited. Alternatively, when the repeat is far from other unfolded regions, the protein is easily degraded [89]. In sum, the presence of specific motifs within the protein sequence regulates the proteasomal degradation in a substrate- and positional-dependent manner. In addition, these findings highlight the importance of the unfolding process for proteasomal degradation.

Another pathway, the ER-associated protein degradation (ERAD) pathway, plays an associated role in protein quality control. This involves the retrotranslocation of errant proteins from the ER into the cytosol for proteasomal degradation [90]. Cholera toxin finds its way into the cell by retrograde trafficking and utilizing ERAD to enter the cytosol. Similarly to typical ERAD substrates, the enzymatic A1 chain of cholera toxin, which is not ubiquitinated itself [91], must first be unfolded by retrotranslocation complex components Derlin-1 and HRD1 [92]. Although the typical substrates exported by ERAD are proteasomally processed, cholera toxin is able to escape this fate by its rapid refolding [91]. In the cytosol, it adopts its native conformation to activate adenylate cyclase by the ADP-ribosylation of the G-protein Gs, resulting in the extreme form of diarrhea that is characteristic of cholera.

In the case of viruses, the ER is used for both entry and replication, as well as for the assembly of infectious viral particles. This can be achieved by hijacking host E3 ligases found in ER. For instance, the human cytomegalovirus (HCMV) takes control over TMEM129, recruited by viral US11, to induce the degradation of MHC-I signaling molecules in a Ub-dependent manner [93] through Derlin1 [94]. Interestingly, rather than using host proteins, the mouse γ-herpesvirus 68 utilizes its own E3 ligase, mK3 [95] to compromise the immune response in infected organisms, exactly like HCMV.

The E3 ligase TRC8, which is essential for US2-mediated MHC-I breakdown, is usurped by US2 from HCMV. In essence, MHC-I undergoes fast polyubiquitination as a result of TRC8 binding to the cytoplasmic tail of US2 [96]. This causes MHC-I to be delivered into the cytoplasm, where it is degraded. While US11-mediated degradation is restricted to MHC-I, US2, unfortunately, also stimulates the downregulation of a number of immunoreceptors to modify cellular motility and immunological signaling [97].

Among its many roles in HIV-1 pathogenesis, the viral protein Vpu counteracts host antiviral responses by the downregulation of the HIV-1 receptor CD4 [98] that is produced de novo in the ER by acting as an adaptor to the Skp1/Cullin1/F-box (SCF) Ub ligase complex through β-TrCP. Concomitantly, the Vpu-β-TrCP complex induces the mono- or polyubiquitination of BST-2 [99,100] to abolish its function of inhibiting the release of infectious viral particles. This complex also promotes the degradation of NF-kB and AP1 [101]. 

Another HIV-1 accessory protein, Vif [102], also functions as an adaptor of cellular ubiquitination pathways. To enhance the probability of successful infection, this virulence factor takes control of the CUL5 E3 ligase by targeting APOBEC3G/F and STAT1/3 to counteract the former [103], and inhibit INF-α-signaling in the case of the latter [104]. The third HIV-1 accessory protein, Vpr, as well as its paralog from HIV-2 and a subset of simian lentiviruses, hijacks the CRL4A (DCAF1) E3 ligase to degrade SAMHD1, a nucleotide triphosphohydrolase that interferes with viral infection [105] and thus enhances viral reverse transcription.

Some HIV proteins compromise the host UPS to mask themselves from recognition and subsequent proteasomal degradation. For example, the integrase (IN) protein, named after its function of inserting the proviral dsDNA into the host genome, is readily proteolyzed in vitro. However, its lifetime in infected cells is significantly prolonged as it hijacks cellular components such as p75 [106] and Ku70 [107] to shield it from recognition by host E3 ligases.

During budding and when leaving the trans-Golgi network, some viruses must ultimately be released from the vesicle upon reaching their final destination. To accomplish this, the herpes simplex virus, HSV-2, hijacks the activity of the Nedd4 family of E3 ligases. By acting as an adaptor protein, the UL56 tegument protein from HSV-2 enhances the ubiquitination of Nedd4 without being ubiquitinated itself [108]. In infected cells, this results in the degradation of Nedd4 in a strictly UL56-dependent manner. More recently, the ORF0 of the varicella–zoster virus, UL42 of HCMV, and U24 of human herpesvirus 6A were shown to bind to Itch, a member of the Nedd4 family, through their PPxY motif, and modulate its activity and reduce its protein level [109].

The HSV-1 E3 ligase ICP0 disrupts components of the so-called promyelocytic leukemia nuclear bodies (PML-NB), which consist of an assembly of about 70 different proteins that prevent the silencing of the viral genome [110]. Specifically, ICP0 targets the degradation of the promyelocytic leukemia protein, which is a critical scaffolding protein required for the recruitment of other associated proteins and the assembly of PML-NB [110]. This E3 ligase and its critical role in the infectious cycle of HSV-1 have recently been comprehensively reviewed [111].

Interestingly, to promote infection, γ-herpesviruses, such as murid herpesvirus-4 (MuHV-4), target Myc, which is essential to the formation and maintenance of germinal center B-cells. In order to stabilize Myc, the viral E3 ligase mLANA catalyzes the attachment of non-canonical Ub chains onto c-Myc, independently of its phosphorylation [112].

In addition to viral E3 ligases and adaptors, the HSV-1 arsenal also encompasses one more kind of effector that affects the host UPS, a DUB UL36. UL36 removes both the K63- and K48-linked polyUb chains of TRAF3 and abrogates the TRAF3 mediation of IFN-β production [113]. Similarly to other effectors, the target list of UL36 is not limited to just a single protein. By deubiquitinating IκBα and thus enhancing its lifetime, the NF-κB activation is inhibited to further dampen host antiviral responses in the DNA sensing pathway [114].

In fact, host proteins involved in antiviral mechanisms are often targeted by different kinds of DUBs. For instance, two papain-like proteases from the severe acute respiratory syndrome coronavirus (SARS-CoV) and the notorious SARS-CoV-2 viruses preferentially target K48-linked polyUb and the ubiquitin-like interferon-stimulated gene 15 protein, respectively [115], both of which are known to act as regulators of the host innate immune pathways. Similarly, pathogenic DUBs expressed by bacteria and viruses (recently reviewed in [116]) target host Ub pathways to compromise the immune response.

Given the important role of the mitochondrial antiviral signaling (MAVS) protein in the antiviral immune response, it is hardly surprising that MAVS represents an attractive target for pathogens. The Orf9b of SARS-CoV usurps the HECT domain E3 ligase AIP4 to trigger the degradation of MAVS, as well as TRAF3 and TRAF 6 [117], which are crucial signaling intermediaries in the antiviral defense. In addition, it also triggers the Ub-dependent proteasomal degradation of dynamin-like protein 1, a host protein involved in mitochondrial fission.

Remarkably, bacterial effectors, such as *Salmonella enterica* AvrA [118] and SSeL [119], also deubiquitinate and impair IκBα degradation in vivo. Another pathogenic function of the latter relies on its ability to remove Ub chains from specific aggregated structures required for *Salmonella* replication, thus preventing their recognition and protecting them from autophagic degradation [120]. More recently, it was suggested that AvrA also decreases Beclin-1 ubiquitination to suppress autophagy [121]. In addition to these DUBs, *Salmonella* produces its own HECT-like E3 ligase, SopA, that targets at least two host E3 ligases, TRIM56 and TRIM65, to promote their degradation and inhibit interferon production [122].

Another human pathogen, *Shigella flexneri*, is known to secrete a range of effectors hijacking host UPS. To date, several *Shigella* E3 ligases have been identified that target several different agents involved in the immune response. For example, a secreted E3 ligase IpaH1.4 decorates the linear Ub chain assembly complex (LUBAC) with K48-chains [123], destining it for degradation. In this way, IpaH1.4 antagonizes the LUBAC-mediated accumulation of M1-linked Ub chains on bacterial surfaces, as well as the recruitment of Optn and Nemo, and abolishes LUBAC-dependent xenophagy [124]. Two other *Shigella* E3 ligases, IpaH7.8 and IpaH0722, have different functions. The former destines the inflammasome inhibitor glomulin for Ub-depended degradation through the activation of caspase-1-mediated cell death [125]; the latter promotes the degradation of TRAF2 to inhibit NF-κB activity in invaded epithelial cells [126]. Host cells have mechanisms that prevent actin-dependent cell-to-cell infection to thwart intracellular bacteria by coating the surface of the invading pathogen with interferon-induced guanylate-binding proteins. The *Shigella* effector, IpaH9.8, interferes with this process through the targeted proteasomal degradation of critical proteins [127]. This clever strategy has been demonstrated for both human cell lines [127] and murine models [128].

In the case of *Legionella pneumophila*, a bacterium causing severe pneumonia known as Legionnaires’ disease, an active host Ub system is required and both E3 ligases and DUBs have been identified among *Legionella*’s ca. 300 effector molecules [129]. AnkB, a F-box domain-containing Ub ligase, directs K48-chain attachment to proteins coating the *Legionella*-containing vacuole (LCV) to induce host proteasomal-degradation of -modified proteins, thus providing an amino acid supply to enhance bacterial proliferation [130].

Fascinatingly, SidE family effectors, including SdeA (Lpg2157), SdeB (Lpg2156), SdeC (Lpg2153), and SidE (Lpg0234), represent a novel type of Ub modification with the ability to modify substrates by means of phosphoribosylated ubiquitination, which is independent of E1 and E2 enzymes [131]. Upon phosphoribosylation on a specific arginine residue, a modified Ub is conjugated by SdeA to serine residues of protein substrates, and this impairs mitophagy, TNF signaling, proteasomal degradation, and other cellular processes [132].

Interestingly, the half-life of pathogenic effectors can be regulated by other effectors, called metaeffectors, in a Ub-dependent manner. *Legionella* LubX and SidH exemplify probably the best-known example of this kind of temporal regulation. Although initially discovered to target host Cdc2-like kinase 1 [133], LubX, a U-box-type E3 ligase, also efficiently promotes the ubiquitination and degradation of SidH [134]. It has been proposed that while SidH is required for the very first phases of infection, it must eventually be degraded by the metaeffector to prevent the death of the host cell [134].

**Figure 1:**
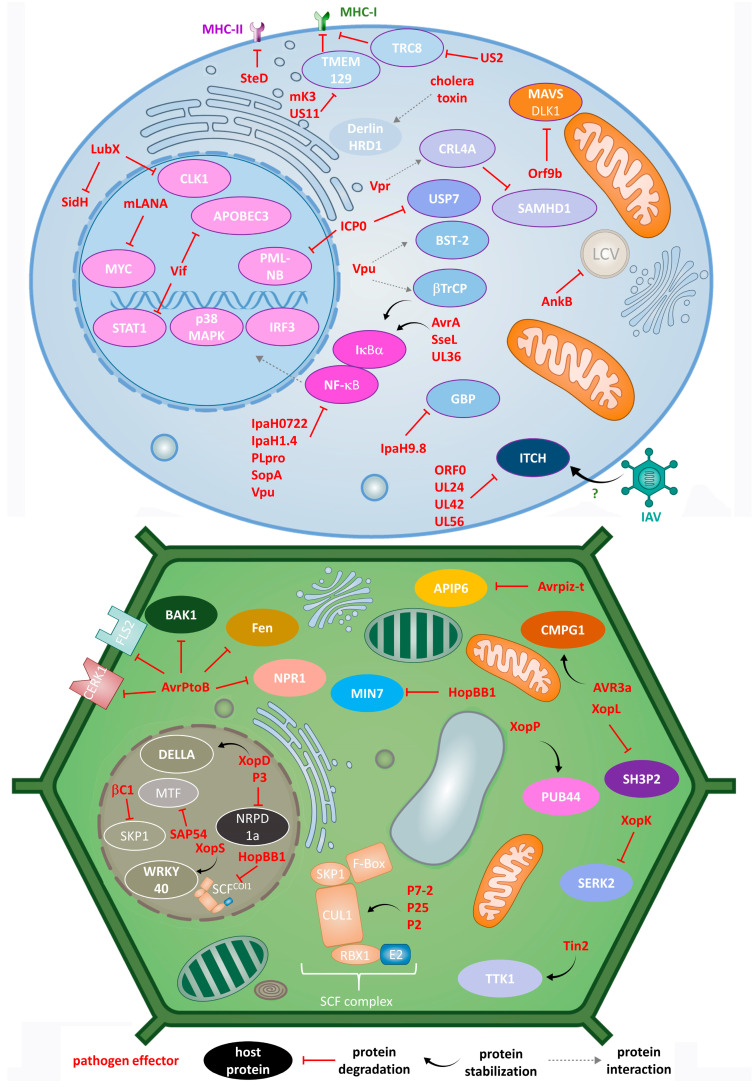
Different modes of action of animal (**top**) and plant (**bottom**) pathogens in the process of hijacking host UPS by effector E3 ligases, DUBs and adaptor proteins.

The discussed impact of pathogens on the animal UPS is presentenced in Figure 1 (top panel).

**Table 1:** Vertebrate pathogens that exploit protein degradation or stabilization by the UPS.

Species	Pathogenic Factor	Host Protein	Targeted Pathway/Effect	Ref.
IAV		Itch	virus endocytosis	[83]
EBV	EBNA-1		abolishing MHC class I-restricted cytotoxic T lymphocyte responses	[85]
cholera	toxin	Derlin-1, HRD1	hijacking the retrotranslocation from ER to the cytosol	[91]
HCMV	US11	TMEM129	degradation of MHC-I signaling molecules	[93]
mouse γ-herpesvirus 68	mK3	TMEM129	degradation of MHC-I signaling molecules	[95]
HCMV	US2	TRC8	degradation of MHC-I signaling molecules	[96]
HIV-1	Vpu	βTrCP	triggering CD4 degradation	[98]
HIV-1	Vpu	BST-2	virion release	[99,100]
HIV-1	Vpu	NF-κB, AP1	suppression of NF-κB activation	[101]
HIV-1	Vif	APOBEC3G/F	antagonization of the APOBEC3 family	[103]
HIV-1	Vif	STAT1/3	inhibition of the production of type I interferons	[104]
HIV-1/2	Vpr	CRL4A (DCAF1)	enhancing lentiviral reverse transcription	[105]
HSV-2	UL56	Nedd4	viral egress	[108]
varicella–zoster virus	ORF0	ITCH	viral egress	[109]
HCMV	UL42	ITCH	viral egress	[109]
human herpesvirus 6A	U24	ITCH	viral egress	[109]
HSV-1	ICP0	PMLNB	abolishing the silencing of the viral genome	[110]
HSV-1	ICP0	USP7	abolishing ICP0 proteasomal degradation	[135]
HSV-1	UL36	TRAF3 IκBα	inhibition of IFN-β production suppression of NF-κB activation	[113,114]
MuHV-4	mLANA	MYC	antagonizing SCF(Fbw7)-mediated proteasomal degradation of Myc	[112]
SARS-CoV2	PLpro	ISG15	antagonizing IRF3 and NF-κB signaling	[115]
SARS-CoV	PLpro	polyUB chains	antagonizing IRF3 and NF-κB signaling	[115]
SARS-CoV	Orf9b	MAVS, TRAF3, TRAF6, DLK1	counteracting antiviral response abolishing mitochondrial fission	[117]
*S. enterica*	SteD	MHC-II	abrogation of antigen presentation	[84]
*S. enterica*	SSeL	IκBα	suppression of NF-κB activation	[119]
*S. enterica*	AvrA	IκBα, β-catenin	suppression of NF-κB activation	[118]
*S. enterica*	AvrA	Beclin-1	suppression of autophagy	[121]
*S. enterica*	SopA	TRIM56, TRIM65	inhibition of the production of type I interferons	[122]
*S. flexneri*	IpaH1.4	LUBAC	suppression of NF-κB activation	[123,124]
*S. flexneri*	IpaH7.8	glomulin	induction of macrophage cell death	[125]
*S. flexneri*	IpaH0722	TRAF2	suppression of NF-κB activation	[126]
*S. flexneri*	IpaH9.8	GBP	protection of bacterial motility	[127]
*L. pneumophila*	AnkB	LCV	providing supply of amino acids	[130]
*L. pneumophila*	SdeA	Ub	impairing mitophagy, TNF signaling, proteasomal degradation of host proteins	[132]
*L. pneumophila*	LubX	Cdc2-like kinase 1	Unknown	[133]
*L. pneumophila*	LubX	*Legionella* SidH	temporal control of infection	[134]

## 6. Plant Pathogens and the UPS

The exploitation of UPS to its own advantage is hardly limited to animal pathogens. In fact, the ubiquitination system has also emerged as a desirable target for plant pathogens. For example, *Xanthomonas campestris*, a bacteria causing disease in tomato and pepper plants, suppresses autophagic turnover in the host cell by utilizing the XopL E3 ligase to promote the Ub-dependent degradation of the autophagy component SH3P2 [136]. Interestingly, XopL belongs to bacterial E3s containing a novel E3 ligase (NEL) domain (recently reviewed in [137]). These proteins are structurally different from eukaryotic E3s and are found in animal and plant pathogens. 

The best-known bacterial effector, however, is *Pseudomonas syringae* AvrPtoB. This effector mimics and displays the activity of an E3 ligase in planta to induce the degradation of NPR1 [138], the key transcriptional regulator of salicylic acid signaling and a master regulator of plant immunity. In fact, the list of substrates targeted by this effector is impressive. Through interaction with many different E2s, its substrates encompass other defense-related proteins, such as BAK1 [139], Fen [140], and FLS2 [141]. In addition, CERK1 is also ubiquitinated, but its degradation occurs in the vacuole [142]. More recently, another fully active bacterial E3 ligase has been described. XopK from *Xanthomonas oryzae pv. oryzae*, responsible for rice bacterial leaf blight, targets a kinase regulating both rice development and immunity, OsSERK2, for proteasomal degradation [143].

Other effectors, not being E3 ligases themselves, act more sophisticatedly in promoting the degradation of host proteins. For instance, HopM1 from *P. syringae* induces the proteasomal degradation of MIN7, a protein known for its role in vesicle trafficking [144]. More information, however, is needed regarding the mechanism of action of this pathogen. By acting as molecular glue, HopBB1, another effector from *P. syringae,* induces the degradation of transcription factor TCP14 through SCF^COI1^ by connecting JAZ3 and promoting virulence [145]. 

A modification of the molecular glue approach was observed in the case of the phytoplasma effector SAP54, which glues MADS-box transcription factors (MTF) to RAD23C and RAD23D, two proteins that shuttle substrates to the 26s proteasome [146]. Another effector from the same pathogenic bacteria, SAP05, bridges distinct classes of plant transcription factors to RPN10 for Ub-independent degradation [147]. Remarkably, these authors reveal that SAP05-binding specificity to RPN10 relies on just two amino acids, one of the few sequence differences between the plant and human von Willebrand factor type A of RPN10 [147].

Some effectors destabilize or stabilize host E3 ligases. For instance, AvrPiz-from *Magnaportheoryzae* mediates the proteasomal degradation of APIP6 to suppress the immunity response in rice [148]. However, it is required by the pathogen to prevent host cell death during infection. In the case of potato blight pathogen *Phytophthora infestans,* this is achieved by the effector AVR3a, which stabilizes the plant E3 ligase CMPG1 [149]. Unlike most U-box proteins, which are widely recognized as negative immune regulators, CMPG1 acts in the opposite way, through stabilization of the suppression of the pathways that result in cell death. 

As one might imagine, enhancing the stability of selected proteins could be beneficial for virulence. Indeed, plant immunity could be weakened upon the successful stabilization of negative regulators. Several examples have been described for *Xanthomonas* effectors. For instance, XopP from *X. oryzae pv. oryzae* prevents ubiquitination of the E3 ligase activity of OsPUB44 [150], a rare representative of U-box proteins that act as positive regulators of immune responses. Once the positive regulation of immune responses is abolished, peptidoglycan- and chitin-triggered immunity is suppressed. Two other effectors, XopD and XopS, act similarly and also abolish the proteasomal degradation of their targets. The former promotes plant disease tolerance by targeting and partially stabilizing DELLA proteins [151]; the latter targets WRKY40, a transcriptional regulator of defense gene expression [152]. In both cases, this leads to enhanced pathogen proliferation, although through different molecular mechanisms.

Proteins that are not directly involved in the UPS can also be protected from proteasomal degradation. For example, the Tin2 effector of a fungus responsible for smut disease in maize, shields the degron found in TTK1 [153]. This protective role stabilizes the active kinase and facilitates fungal proliferation. 

Viruses have evolved ways to induce the expression of the desired endogenously encoded E3 genes. For instance, the P3 protein encoded by the Rice grassy stunt virus induces U-box type E3 ligase, P3IP1, leading to the Ub-dependent degradation of rice OsNRPD1a, the largest subunit of plant-specific RNA polymerase IV [154]. Another previously introduced strategy is to interfere with E3 ligases. In the case of viruses, it has been demonstrated that βC1 from cotton leaf curl Multan virus [155], and P7-2 [156] and P25 [157], both encoded by rice black-streaked dwarf virus, all target SKP1, a core subunit of the SCF complex promoting protein degradation. The rice dwarf virus protein P2 targets auxin signaling and inhibits SCF-mediated OsIAA10 proteasomal degradation. This is achieved by blocking protein–protein interactions between OsIAA10 and OsTIR and enhancing viral infection [158].

Fascinatingly, even in the case of *Agrobacterium tumefaciens*, a plant pathogen that is utilized worldwide for plant genetic engineering [159], proteasomal degradation is pivotal for efficient transformation. *Agrobacterium* virulence proteins, together with the host SKP1/culin/F-box (SCF)–E3 ligase complex, target the degradation and release of the T-DNA, and this allows for its integration into the plant genome [160] and the heterologous expression of the desired gene product. The plant pathogens discussed above that interfere with host UPS are summarized in Table 2.

The discussed impact of pathogens on plant UPS is presentenced in Figure 1 (bottom panel).

## 7. Conclusions

In summary, a single protein can undergo proteasomal degradation via different, distinct mechanisms. The latest discoveries highlight the different functions of various Ub-chains and the critical role of the unstructured regions of targeted proteins. Remarkably, pathogenic bacteria and viruses take control of Ub-dependent or independent, 26S or 20S proteasome-dependent protein degradation at different stages of their development to promote infection. As discussed, several protein-degradation mechanisms can be targeted by different effectors, such as E3 ligases, DUBs, and molecular glues, originating from the same pathogen. It is safe to assume that more examples will be discovered in the future as we elucidate the role of hundreds of currently poorly understood effectors. On the one hand, microorganisms that lack their own UPS have the ability to hijack the host UPS to degrade immunologically relevant host machinery or the ability to protect their own proteins from degradation, which is worrying. On the other hand, this presents the possibility of developing new drugs directed towards the different mechanisms of action of these pathogenic effectors.

## Figures and Tables

**Table 2:** Plant pathogens interfering specifically with protein stabilization/degradation with host UPS.

Species	Pathogenic Effector	Host Protein	Targeted Pathway/Effect	Ref.
*X. capestris*	XopL	SH3P2	abolishing autophagy	[136]
*P. syringae*	AvrPtoB	NPR1	deregulation of plant immunity	[138]
*P. syringae*	AvrPtoB	BAK1	deregulation of plant immunity	[139]
*P. syringae*	AvrPtoB	Fen	deregulation of plant immunity	[140]
*P. syringae*	AvrPtoB	FLS2	deregulation of plant immunity	[141]
*P. syringae*	AvrPtoB	CERK1	enhancing bacterial virulence	[142]
*P. syringae*	HopM1	MIN7	manipulation of vesicle trafficking	[144]
*P. syringae*	HopBB1	SCF^COI1^	promotion of host transcriptional repressor degradation to regulate phytohormone responses and virulence	[145]
*M. oryzae*	Avrpiz-t	APIP6	suppression of PAMP-triggered immunity	[148]
*P. infestans*	SAP54	MTF	induction of insect colonization	[146]
*P. infestans*	AVR3a	CMPG1	Prevention of cell death upon infection	[149]
*X. oryzae*	XopP	PUB44	suppression of PAMP-triggered immunity	[150]
*X. oryzae*	XopK	SERK2	deregulation of plant immunity	[143]
*X. oryzae*	XopD	DELLA	induction of plant disease tolerance	[151]
*X. oryzae*	XopS	WRKY40a	deregulation of plant immunity	[152]
*U. maydis*	Tin2	TTK1	enhancing fungal proliferation	[153]
Rice grassy stunt virus	P3	P3IP1	degradation of OsNRPD1a, inducing hypomethylation of downstream genes	[154]
Cotton leaf curl Multan virus	βC1	SKP1	enhancing virus DNA accumulation	[155]
Black streaked dwarf virus	P7-2	SCF-E3 ligase	possible role in the gibberellin signaling pathway	[156]
Beet necrotic yellow vein virus	P25	SCF-E3 ligase	enhancing virus pathogenicity	[157]
Rice dwarf virus	P2	SCF-E3 ligase	enhancing viral infection	[158]
*A. tumefaciens*		SCF-E3 ligase	release of T-DNA	[160]
